# Pathogen-Host Interaction Repertoire at Proteome and Posttranslational Modification Levels During Fungal Infections

**DOI:** 10.3389/fcimb.2021.774340

**Published:** 2021-12-02

**Authors:** Yanjian Li, Hailong Li, Tianshu Sun, Chen Ding

**Affiliations:** ^1^ College of Life and Health Sciences, Northeastern University, Shenyang, China; ^2^ NHC Key Laboratory of AIDS Immunology (China Medical University), National Clinical Research Center for Laboratory Medicine, The First Affiliated Hospital of China Medical University, Shenyang, China; ^3^ Medical Research Centre, State Key Laboratory of Complex Severe and Rare Diseases, Peking Union Medical College Hospital, Chinese Academy of Medical Science, Beijing, China; ^4^ Beijing Key Laboratory for Mechanisms Research and Precision Diagnosis of Invasive Fungal Diseases, Beijing, China

**Keywords:** fungal pathogens, proteome, mass spectrometry, virulence factors, host–pathogen interaction, posttranslational modification

## Abstract

Prevalence of fungal diseases has increased globally in recent years, which often associated with increased immunocompromised patients, aging populations, and the novel Coronavirus pandemic. Furthermore, due to the limitation of available antifungal agents mortality and morbidity rates of invasion fungal disease remain stubbornly high, and the emergence of multidrug-resistant fungi exacerbates the problem. Fungal pathogenicity and interactions between fungi and host have been the focus of many studies, as a result, lots of pathogenic mechanisms and fungal virulence factors have been identified. Mass spectrometry (MS)-based proteomics is a novel approach to better understand fungal pathogenicities and host–pathogen interactions at protein and protein posttranslational modification (PTM) levels. The approach has successfully elucidated interactions between pathogens and hosts by examining, for example, samples of fungal cells under different conditions, body fluids from infected patients, and exosomes. Many studies conclude that protein and PTM levels in both pathogens and hosts play important roles in progression of fungal diseases. This review summarizes mass spectrometry studies of protein and PTM levels from perspectives of both pathogens and hosts and provides an integrative conceptual outlook on fungal pathogenesis, antifungal agents development, and host–pathogen interactions.

## Introduction

Fungal pathogenic diseases that cause high mortality and morbidity are increasing in prevalence globally, coincident with accelerating numbers of patients with COVID-19, HIV infection, and organ transplants (Hurtado et al., 2019; Stone et al., 2019; Heard et al., 2020; Hoving et al., 2020; Song G. et al., 2020; Yoon et al., 2020; Rawson et al., 2021). Furthermore, invasive fungal infections are intractable because of long treatment cycles and high probability of relapse (Ecevit et al., 2006). Common human pathogenic fungi, including *Candida albicans*, *Aspergillus fumigatus*, and *Cryptococcus neoforman*s, are opportunistic pathogens that are always associated with host immune status (Alhumaid et al., 2021; Pasquier et al., 2021; Singh et al., 2021). To invade a host and replicate and spread, pathogens need to obtain host resources, such as a carbon source, proteins, and lipids, and avoid or take advantage of host defense mechanisms. Pathogens have evolved a variety of virulence factors, such as biofilms, capsules, morphologic transformations, and kinase systems, to facilitate infection (Ding and Butler, 2007; Wang et al., 2012; Do et al., 2018; Suo et al., 2018; Lee et al., 2019; Vu et al., 2019). In response to fungal attack, hosts alter the microenvironment and activate the immune system by modifying body temperature, oxidation levels, and metal contents, limiting nutrients, and increasing levels of inflammatory factors and immune cells (Hu et al., 2008; Butler et al., 2009; Kronstad et al., 2011; Kronstad et al., 2012; Saikia et al., 2014; Rohatgi and Pirofski, 2015; Hole and Wormley, 2016; Ballou and Johnston, 2017; Hansakon et al., 2019; Sun et al., 2019).

Pathogens and hosts require rapid modulation of virulence and defense mechanisms, which is a conclusion validated by many different biological technologies (Butler et al., 2009; Kronstad et al., 2011; Kronstad et al., 2012). For example, alterations at the *C. neoformans* and host (mouse and *Macaca fascicularis*) axis were monitored with transcriptome technology (Li H. L. et al., 2019). Genes were expressed to counter fungal invasion that were involved in immune and inflammatory responses, osteoclastogenesis (in particular, osteoclastogenesis-associated gene (*OC-STAMP*)), and insulin signaling. The fungus responded rapidly by activating metal sequestration, dampening sugar metabolism, and changing cell morphology to increase its survival in the host (Li H. L. et al., 2019). However, important aspects of complex host–pathogen interactions are addressed differently by different techniques (Jacobsen et al., 2018; Li H. L. et al., 2019; Li et al., 2020).

**Figure 1:**
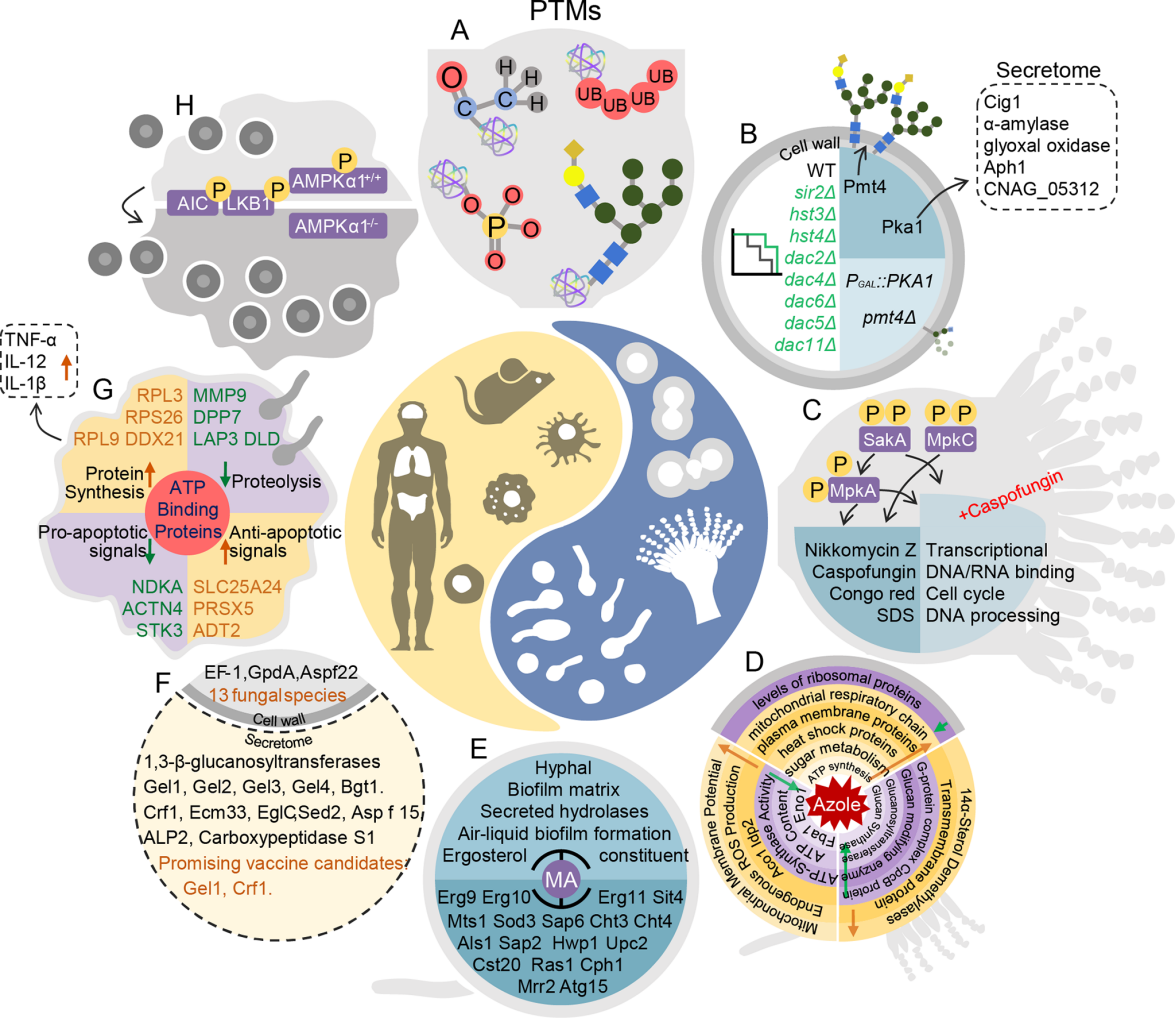
Pathogen–host interaction repertoire at proteome and posttranslational modification levels during fungal infections. **(A)** Posttranslational modifications in fungal pathogenesis. **(B)** In *Cryptococcus neoformans*, deacetylases Sir2, Hst3, Hst4, Dac2, Dac6, Dac4, Dac5, and Dac11 are all essential for pathogenesis. Knockout of *PMT4* decreases protein mannosylation inefficiency. In the *PGAL7::PKA1* strain, expression of 61 secretome proteins changes, including that of Cig1, α-amylase, glyoxal oxidase, Aph1, and CNAG_05312. **(C)** In *Aspergillus fumigatus*, SakA^HOG1^, MpkC, and MpkA are phosphorylated. *sakAΔ* and *mpkCΔsakAΔ* are more sensitive to caspofungin and nikkomycin Z, congo red, and sodium dodecyl sulfonate (SDS). In *mpkAΔ* and *sakAΔ* treated with high doses of caspofungin, decreases occur in DNA/RNA binding, cell cycle control, and DNA processing pathways. **(D)** Top: In response to fluconazole, in *Cryptococcus neoformans*, ribosomal proteins decrease and heat shock proteins, plasma membrane proteins, and proteins involved in glucose metabolism, ATP synthesis, and mitochondrial respiratory chains increase over time. Left: When *Candida albicans* is treated with fluconazole, mitochondrial membrane potential, endogenous reactive oxidative species production, and Aco1 Idp2 are up-regulated. Right: When *Aspergillus fumigatus* is exposed to itraconazole, 14α-sterol demethylases and transmembrane proteins are up-regulated, and G-protein complex, glucan modifying enzyme, glucanosyltransferase, and glucan synthase are down-regulated. **(E)** In *Candida albicans*, MA inhibits hyphae, biofilm matrix, secreted hydrolases, air–liquid biofilm formation, and ergosterol constituents by regulating Erg9, Erg10, Erg11, Sit4, Mts1, Sod3, Sap6, Cht3, Cht4, Als1, Sap2, Hwp1, Upc2, Cst20, Ras1, Cph1, Mrr2, and Atg15. **(F)** In the intracellular proteome and secretome of 13 fungi, cell extracts consist of EF-1, GpdA, and Aspf22. Secretion consists of 1,3-beta glucanosyltransferases, including Gel1, Gel2, Gel3, Gel4, Bgt1, Crf1, Ecm33, EglC, Sed2, Asp f15, ALP2, and carboxypeptidase S1. Gel1 and Crf1 screen as promising vaccine candidates. **(G)** ATP-binding proteins are enriched in macrophages infected with *Candida albicans*. Anti-apoptotic proteins PRDX5, SLC25A24, and ADT2 increase, whereas pro-apoptotic proteins NDKA, ACTN4, and ST3 decrease. Ribosomal proteins RPL9, RPS26, and RPL3 increase. Proteolysis-associated proteins MMP9, DPP7, LAP3, and DLD decrease. In addition, secretion of TNF-α, IL-12 and IL-1β increase. **(H)** Deletion of AMPKα1 in monocytes leads to resistance to *Cryptococcus neoformans* colonization in mice.

Over past decades, application of MS-based proteomics has expanded rapidly, especially in studies of proteomes and posttranslational modifications (PTMs), such as acetylation, phosphorylation, succinylation, and crotonylation (**Figure 1A**) (Aggarwal et al., 2021). Application in studies of microbiological pathogenesis and interactions between pathogens and hosts has led to the discovery of many novel mechanisms of host–fungus interactions (Toor et al., 2018; Khan et al., 2019; Li Y. et al., 2019; Zamith-Miranda et al., 2019; Bruno et al., 2020; Machata et al., 2020; Thak et al., 2020; Zhou et al., 2021). Establishing connections between proteomic profiles and fungal infection processes is critical in characterizing disease pathophysiology, developing candidate therapies, and predicting clinical outcomes.

This review will focus on the applications of MS-based proteomics to examine protein and PTM levels from perspectives of both pathogens and hosts, give a comprehensive opinion and novel outlook on fungal pathogenesis, antifungal therapy, and host–pathogen interactions.

## Proteomic Profiles of Fungal Pathogen Responses to Stress

Proteomics can contribute to understanding variations in global protein expression in fungal pathogens under stress. Plasticity in fungal pathogen response to different host microenvironments is important for successful infection. Maintaining oxidative homeostasis is a critical strategy as fungal pathogens adapt to their hosts (**Table 1**). *Aspergillus fumigatus* tolerates hypoxic conditions in lung infections, and therefore, differentially expressed proteins under hypoxic treatment can reflect fungal virulence performance (Warn et al., 2004; Tarrand et al., 2005; Willger et al., 2008). Proteins involved in glycolysis, tricarboxylic acid(TCA) cycle, oxidative phosphorylation, ergosterol biosynthesis, metals metabolism, secondary metabolism, and generation of nitrosative stress are differentially expressed under hypoxic conditions in *A. fumigatus* (Vodisch et al., 2011; Barker et al., 2012). Metals are widely known to participate in stress resistance in fungi (Ding et al., 2011; Samanovic et al., 2012; Ding et al., 2013; Ding et al., 2014a; Ding et al., 2014b; Sun et al., 2014; Do et al., 2018; Li et al., 2019a). In *A. fumigatus*, additional oxidative stress response is related to iron availability (Kurucz et al., 2018). Furthermore, starvation and high concentrations of metal ions, such as iron and copper, are challenges from the natural environment and host (Ding et al., 2014a; Sun et al., 2014; Li et al., 2019b). In microsomal proteome analysis of *A. fumigatus*, 231 proteins were significantly differentially expressed between iron-rich and iron-depleted conditions, which included siderophore transporters, indicating that microsomal proteins were associated with iron-depleted conditions (Moloney et al., 2016). In another study, iron-responsive microsomal protein MirC was associated with maintenance of iron homeostasis in *A. fumigatus*, which was consistent with the increased abundance of siderophore biosynthetic enzymes in *mirCΔ* (Mulvihill et al., 2017). Protein phosphatase PpzA, an iron assimilation factor, influences the pathogenicity of *A. fumigatus* by reducing secondary metabolites under iron starvation (Manfiolli et al., 2017). To investigate iron homeostasis, proteomic analysis based on cross-linked tandem affinity purification coupled with MS was also performed in *C. albicans*, and Fra1, Bol2/Fra, Sfu1, and Hap43 were found to interact with iron homeostasis regulator monothiol glutaredoxin 3 (Alkafeef et al., 2020). Similarly, proteomics was used to study the role of copper homeostasis in *C. neoformans* (Sun et al., 2021). Under copper stress, the proteasome pathway was up-regulated and the ribosomal pathway down-regulated. In addition, the ubiquitination level of whole proteins was up-regulated under copper stress, and a growth defect could be restored by inhibiting the proteasome pathway (Sun et al., 2021).

**Table 1:** Summary of proteomic studies in fungal pathogens.

Functions	Pathogens	Description	Reference
Proteomic Profiles of Fungal Pathogen Responses to Stress	*A. fumigatus*	Proteomic studies have found that some key pathways differ under stresses, including hypoxic conditions, oxidative stress, iron-rich conditions, iron-depleted conditions, and osmotic stress.	(Vodisch et al., 2011; Barker et al., 2012; Ding et al., 2014a; Sun et al., 2014; Moloney et al., 2016; Manfiolli et al., 2017; Li et al., 2019a; Silva et al., 2020)
	*C. albicans*	Proteomic analysis was performed to identify the special role of monothiol glutaredoxin 3 in iron homeostasis regulation.	(Alkafeef et al., 2020)
	*C. neoformans*	Studies have used proteomics to examine pathway responses to copper stress and high-temperature stress.	(Martinez Barrera et al., 2020; Sun et al., 2021)
Proteomic Profiles of Virulence Gene-Edited Fungal Strains	*C. albicans*	Proteomic analysis was used to identify protein components of plasma, and specific functions of regulator involved in cell wall formation, morphogenesis, cell differentiation, and pathogenicity.	(Cabezon et al., 2009; Lee et al., 2010; Santi et al., 2014)
	*C. neoformans*	Proteomic profiles were performed to analyze biofilm, capsule formation and cell growth.	(Olson et al., 2007; Santi et al., 2014; Geddes et al., 2016; Bruni et al., 2017)
Posttranslational Modifications in Fungal Pathogenesis	*C. neoformans*	Kinases involved in the cell cycle, metabolic processes, and virulence adjustment were detected in phosphoproteomic analysis.	(Selvan et al., 2014)
	*A. fumigatus*	Phosphorylation modified proteins were detected under Congo red and sorbitol induce and caspofungin treatment.	(Mattos et al., 2020a; Mattos et al., 2020b)
	*C. neoformans, C. albicans*	Large abundant of ubiquitin proteasome pathway (UPP)-related proteins were identified by proteomic studies.	(Atir-Lande et al., 2005; Liu and Xue, 2014; Geddes et al., 2016)
	*C. neoformans, C. albicans*, and *A. fumigatus*	Function of histone deacetylases were analyzed by proteomic studies and acetylomes of baker’s yeast and three human fungal pathogens were compared.	(Arras et al., 2017; Li Y. et al., 2019)
Secretomic Profiles of Fungal Pathogens	*C. neoformans*	Main component proteins of extracellular vesicles and extracellular proteome were analyzed by MS.	(Rodrigues et al., 2008; Vu et al., 2014; Wolf et al., 2014; Vargas et al., 2015; Bielska and May, 2019)
	*C. albicans*	Protein composition of EVs is associated with pathogenesis, cell organization, carbohydrate and lipid metabolism, branching and biofilm formation.	(Thomas et al., 2009; Vargas et al., 2015; Wolf et al., 2015)
	*A. fumigatus*	Proteomic analysis was performed to determine the expression of secreted proteases in *ptrtΔ*, *xprgΔ*, and *ptrt/xprgΔ*.	(Shemesh et al., 2017)
Drug Action and Pharmacological Effects on Proteomic Profiles	*C. gattii*	In a time-course proteomic analysis was performed during fluconazole treatment.	(Chong et al., 2012)
	*C. albicans*	Proteomic analysis revealed a synergistic mechanism of fluconazole and berberine against fluconazole-resistance.	(Xu et al., 2009)
	*A. fumigatus*	Proteomic analysis was performed in cells treated with itraconazole.	(Gautam et al., 2016)
	*C. glabrata*	Proteomic analysis was performed in fluconazole-induced resistant strains.	(Samaranayake et al., 2013)
Vaccine Screening for Fungal Pathogens	*C. neoformans, A. fumigatus, C. gattii*	Secreted and cell wall-bound proteins were identified by Immunoblot-MS analyses.	(Eigenheer et al., 2007; Young et al., 2009; Kumar et al., 2011; Chaturvedi et al., 2013; Martins et al., 2013; Virginio et al., 2014)
	13 fungal species	Highly conserved secreted and surface proteins from were identified.	(Champer et al., 2016)

Responses of fungal pathogens to high temperatures and osmotic pressures are also key factors affecting infection, but only a few studies have used proteomics to examine those responses. Potential binding partners of septin Cdc10 in *C. neoformans* were scanned using Immunoprecipitation(IP)-proteome analysis in order to explain the outstanding protective effect of Cdc10 against high-temperature stress (Martinez Barrera et al., 2020). In *A. fumigatus*, Sln1p, Msb2p, and Opy2p, upstream sensors of the high-osmolarity glycerol(HOG) pathway, affect osmotic stress response, carbohydrate metabolism, and protein degradation (Silva et al., 2020). Additional proteomic analyses investigating fungal pathogen response to stress should be performed in the future to develop new clinical treatments for fungal disease.

## Proteomic Profiles of Virulence Gene-Edited Fungal Strains

Virulence factors such as capsules, melanin, morphology, biofilm formation, virulence genes, plasma membranes, and cell wall maintenance have critical roles in fungal pathogen invasion (Crabtree et al., 2012; Dambuza et al., 2018; Mukaremera et al., 2018). Proteomics is a reliable approach to explore regulatory functions of virulence factors (**Table 1**). For example, in the yeast-to-hyphal transition factor *CaKEM1* mutant strain of *C. albicans*, proteomic analysis was used to identify hyphae-specific genes that were regulated (Lee et al., 2010). Proteomic profiles were compared between biofilm cells and planktonic cells of *C. neoforman*s in order to better understand the biofilm lifestyle, and proteins involved in oxidation–reduction, proteolysis, transport, translation, and energy acquisition mode were enriched (Santi et al., 2014). In an analysis of protein components of plasma membranes in *C. albicans*, 12 glycosylphosphatidylinositol(GPI)-anchored membrane proteins were associated with cell wall maintenance and virulence (Cabezon et al., 2009).

Proteomics can also help detect plasma membrane and cell-wall regulate genes associated with mutant-specific protein expression. The protein O-mannosyltransferase (Pmt protein) is associated with the cell wall and morphogenesis. Knockout of *PMT4* in *C. neoformans* decreases expression of wall component proteins and leads to protein mannosylation inefficiency (**Figure 1B**) (Olson et al., 2007). Proteomic analysis also determined that *PKA* regulates capsule formation through a ubiquitin–proteasome pathway in *C. neoformans* (Geddes et al., 2016) (**Figure 1B**). Secretomic analysis of a *PKA1* expression-suppression strain revealed five biomarkers of infection, including definitive virulence factors Cig1 and Aph1 (**Figure 1B**) (Geddes et al., 2015). On the basis of proteomics, the F-box protein Fbp1 affects *C. neoformans* survival in macrophages by regulating inositol sphingolipid biosynthesis (Liu and Xue, 2014). The functions of Gib2 are vital in cell growth, differentiation, and pathogenicity. A two-dimensional echocardiography(2DE)-MS analysis of *gib2Δ* showed that Gib2 was linked to ribosomal biogenesis, protein translation, and stress responses in *C. neoformans* (Bruni et al., 2017). Virulence factors are potential targets for new antifungal drugs, and thus, further investigations of virulence genes associated with cell walls, plasma membranes, and the cell cycle are needed.

## Posttranslational Modifications in Fungal Pathogenesis

In evaluating the virulence of fungal pathogens, epigenetic modifications are a more direct and rapid response to stress. Epigenetic modifications that have received wide attention include PTMs, such as phosphorylation, ubiquitination, and acetylation (**Table 1**) (Aggarwal et al., 2021; Zhang Y. et al., 2021). Phosphorylation regulates kinase pathways during fungal infection. For example, Hog1 is a ubiquitous MAPK enzyme in fungi that responds to external stimuli such as temperature, osmotic pressure, and oxidative damage. Hog1 is phosphorylated in *C. neoformans* serotype D but is dephosphorylated in serotype A under stress (Bahn et al., 2006). Forty-five kinases involved in the cell cycle, metabolic processes, and virulence adjustment were detected in phosphoproteomic analysis in *C. neoformans*, and the kinases included protein kinase C, Bck1, Mkk2, and Mpl1 (Selvan et al., 2014). Similar studies have been conducted on *A. fumigatus.* Knockout of Hog1 homologous genes *SAKA* and *MPKC* in *A. fumigatus* increased sensitivity to osmotic and oxidative stress and cell damages. Congo red and sorbitol induce MpkC phosphorylation modification in *A. fumigatus* (**Figure 1C**) (Bruder Nascimento et al., 2016). Phosphorylation modification was also detected on p38 (CMGC/MAPK/p38/Hog) (Mattos et al., 2020b). Low expression of phosphorylase in *sakAΔ*, *mpkCΔ*, and *mpkC/sakAΔΔ* indicates that phosphorylation is essential for MpkA to maintain cell walls (**Figure 1C**) (Mattos et al., 2020b). Meanwhile, with caspofungin treatment, phosphorylated proteins included transcription factors, protein kinases, and cytoskeletal proteins. In s*akAΔ*, *mpkAΔ*, and *mpkA/sakAΔΔ*, phosphorylation levels of metabolic and transcriptional regulatory proteins, DNA/RNA binding proteins, and cell cycle control proteins are down-regulated (**Figure 1C**). When treated with caspofungin, phosphorylation levels of protein kinases A (PKA) regulatory subunit, protein kinases C (PKC phosphorus transcription factor AtfA/AtfB/AtfD), and transcription factor ZipD were down-regulated (Mattos et al., 2020a). Therefore, regulation of the MAPK pathway by affecting posttranslational modifications is a potential target for new drugs.

As described above, stress response pathways in fungi facilitate survival and adaptation during infection. Geddes et al. (2016) used proteomics to identify the effect of *PKA1* mutation on intracellular proteins in *C. neoformans* and 302 differentially expressed proteins were identified. Ribosome and translation-related proteins were the most abundant in protein–protein interactions, whereas ubiquitin proteasome pathway (UPP)-related proteins were the second most abundant (Geddes et al., 2016). UPP damage is associated with pathogenesis of a variety of neurodegenerative diseases, including Alzheimer’s, Parkinson’s, and Huntington’s, suggesting that UPP plays a critical role in maintaining cellular protein homeostasis (Huang and Figueiredo-Pereira, 2010; Nijholt et al., 2011). The SCF (Skp1, Cullins, and F-box proteins) E3 ubiquitin ligases are involved in various biological processes in pathogenic fungi. In *C. neoformans*, Liu et al. (2011) demonstrated that SCF^Fbp1^E3 ubiquitin ligase is indispensable during infection. In an *FBP1* knockout strain, fungal pulmonary burden and proliferation ability in macrophages decrease, resulting in inability to migrate in a host (Liu and Xue, 2014). Fbp1 also helps mediate sexual reproduction in *C. neoformans* (Liu et al., 2011). In *C. albicans*, SCF E3 ubiquitin ligase helps regulates mycelial morphology (Butler et al., 2006). For example, SCF^Cdc4^ is involved in negative regulation of fungal filaments (Atir-Lande et al., 2005), whereas SCF^Grr1^ is involved in negative regulation of pseudomycelia (Butler et al., 2006). In *Aspergillus nidulans*, SCF^GrrA^ is involved in meiosis and sexual sporogenesis (Krappmann et al., 2006). These results indicate that the ubiquitin–proteasome pathway is involved in cell cycle regulation and fungal transformation.

Autophagy also helps to maintain protein homeostasis in cells. Autophagy is a response to various environmental stresses, such as nutritional deficiencies and hypoxia (Shliapina et al., 2021). Many studies show that induction of autophagy depends primarily on the serine/threonine protein kinase TOR regulating the phosphorylation level of the core Atg protein (Jung et al., 2010; Paquette et al., 2018; Wang and Zhang, 2019). In yeast, TOR regulates the phosphorylation level of Atg13, resulting in a decrease in the affinity between Atg1 and its binding proteins, and subsequently inhibits the initiation of autophagy under nutrient-rich conditions (Kawamata et al., 2008; Jung et al., 2010). In addition, several Atg proteins undergo changes in acetylation state, indicating that acetylation modification is very important in the regulation of autophagy (Lee and Finkel, 2009; Yi et al., 2012; Banreti et al., 2013). Acetylation is also involved in many other biological processes and cellular activities of fungi, including host adaptability, genome stability, production of virulence factors, synthesis of secondary metabolites, and fungal drug resistance (Lee et al., 2009; Wurtele et al., 2010; Lu et al., 2012; Lamoth et al., 2014; Brandao et al., 2015; Freire-Beneitez et al., 2016). In *C. neoformans*, deletion of the histone deacetylases SIR2, HST3, and HST4 significantly altered the epigenetic landscape and virulence (Arras et al., 2017). Essential in the pathogenesis in *C. neoformans* is the deacetylases Sir2, Hst3, Hst4, Dac2, Dac6, Dac4, Dac5, and Dac11 (**Figure 1B**) (Li Y. et al., 2019). Li Y. et al. (2019) also compared acetylomes of baker’s yeast and three human fungal pathogens (*C. neoformans*, *C. albicans*, and *A. fumigatus*). Thus, the acetylation motifs of fungal pathogens participate in mediating pathogenicity and therefore are subject to selective evolution (Li Y. et al., 2019). The study provides a reference for further investigations of the evolution of protein translational modifications in pathogenic fungi.

## Secretomic Profiles of Fungal Pathogens

Extracellular vesicles (EVs) deliver secretory proteins into a host. In *C. neoformans*, 76 proteins in EVs are linked to virulence and protection against oxidative stress during infection (Rodrigues et al., 2008). With increased technological sensitivity, another 147 proteins were identified as main component proteins in EVs (Wolf et al., 2014). Composition of EV proteins is closely associated with virulent phenotypes (Vargas et al., 2015; Bielska and May, 2019). *C. neoformans* needs to penetrate the blood brain barrier (BBB) in order to invade the central nervous system, and vesicles play an important role in that process (Vu et al., 2014). A secreted metalloproteinase, Mpr1, identified in extracellular proteome analysis was found to play an important role in breaching the BBB (Vu et al., 2014). In *C. albicans*, protein composition of EVs is associated with pathogenesis, cell organization, carbohydrate and lipid metabolism, and response to stress (Vargas et al., 2015). For example, a *VPS4* mutation in *C. albicans* leads to reductions in normally secreted proteins, which may associated with altered branching and biofilm formation (Thomas et al., 2009). Defects in lipid biosynthetic genes *CHO1*, *PSD1*, and *PSD2* lead to significant changes in the exponential cargo of EVs (Wolf et al., 2015). Mutation in the cell wall protein-encoding gene *DSE1* leads to a lack of chitin biosynthesis protein Chs5 and stimulates the expression of the cell wall degrading-related protein glucoamylase 1 (Zohbi et al., 2014). In *A. fumigatus*, the release of extracellular proteases to degrade host structures is also an important fungal virulence factor. Transcription factors XprG and PrtT regulate extracellular proteolysis. Proteomic analysis was performed to determine the expression of secreted proteases in *ptrtΔ*, *xprgΔ*, and *ptrt/xprgΔ*, and the expression levels of 24 proteases, 18 glucanases, 6 chitinases, and 19 allergens decreased by two to fivefold (Shemesh et al., 2017). Because secretory proteins affect fungal virulence from several aspects, secretomes of pathogenic fungi are currently a hot topic of research.

## Drug Action and Pharmacological Effects on Proteomic Profiles

Fluconazole, voriconazole, and itraconazole are widely used in prophylactic and maintenance therapies (Day et al., 2013; Rajasingham et al., 2017). In a time-course proteomic analysis of *Cryptococcus gattii* during fluconazole treatment, most ribosomal proteins decreased, whereas mitochondrial respiratory chain, plasma membrane, and heat shock proteins and those associated with sugar metabolism and ATP synthesis increased (**Figure 1D**) (Chong et al., 2012). In *C. albicans*, proteomic analysis revealed a synergistic mechanism of fluconazole and berberine against fluconazole-resistance. Mitochondrial membrane potential, endogenous reactive oxygen species (ROS) production, and the TCA cycle (Aco1, Idp2) were up-regulated; whereas ATP content, ATP-synthase (complex V) activity, and glycolysis (Fba1, Eno1) were down-regulated (**Figure 1D**) (Xu et al., 2009). In *A. fumigatus* cells treated with itraconazole, abundances of 14α-sterol demethylases, transmembrane proteins, G-protein complexes, glucan modifying enzymes, glucanosyl transferases, and glucan synthases were altered (**Figure 1D**) (Gautam et al., 2016). Eight fluconazole-induced resistant strains of *Candida glabrata* changed in expression of proteins associated with bud formation and metallothionein production (Samaranayake et al., 2013).

Some natural compounds are effective antifungal agents, and proteomic analysis has been used to explore their affected targets and mechanisms of control. Myristic acid (MA) and oleic acid affect biofilm formation and virulence of *C. albicans* by regulating ergosterol synthesis, sphingolipid metabolism, and lipase production proteins (**Figure 1E**) (Prasath et al., 2019; Muthamil et al., 2020). In *A. fumigatus* exposed to cis-9-hexadecenal, PKS enzymes are up-regulated and the 1,8-dihydroxynaphthalene-melanin biosynthesis pathway is down-regulated. Induced oxidative stress is also an important mechanism of candidate antifungal agents. N-chlorotaurine inhibits conidial and mycelial growth in *A. fumigatus* by up-regulating the oxidative stress response (Sheehan et al., 2019). Atorvastatin has treatment potential because it induces oxidative stress and alters membrane permeabilit*y* in *A. fumigatus* (Ajdidi et al., 2020). Such novel antifungal drugs are welcomed additions in clinical therapy.

## Vaccine Screening for Fungal Pathogens

Extracellular proteins participate in fungal pathogenesis as immunoreactive antigens (Zhang L. et al., 2021). In an analysis of secreted and cell wall-bound proteins in *C. neoformans*, extracellular proteins possessed immunogenicity and proteolytic ability for the glycosylphosphatidylinositol-anchored proteins that were recruited to the cell wall (Eigenheer et al., 2007). Immunoblot-MS analyses have been conducted with fungal pathogens to identify diagnostic markers or candidate antigens for development of vaccines and immunotherapy (Young et al., 2009; Kumar et al., 2011; Chaturvedi et al., 2013; Martins et al., 2013; Virginio et al., 2014). Highly conserved secreted and surface proteins from 13 fungal species were identified, including the following 1,3-β-glucanosyltransferases: Gel1, Gel2, Gel3, Gel4, Bgt1, Crf1, Ecm33, EglC, Sed2, Asp f15, ALP2, and carboxypeptidase S1. Gel1 and Crf1 were screened as promising vaccine candidates (**Figure 1F**) (Champer et al., 2016). Vaccines are widely used to prevent bacterial and viral infections; however, some obstacles impede vaccine development for fungal pathogens. For example, β-1,3-D-glucan, a key component of fungal cell walls, is poorly immunogenic (Armstrong-James et al., 2017). The sensitivity and high throughput of mass spectrometry have been improved, creating unprecedented opportunities to exploit fungal vaccine. However, the fungal vaccines are still on the way.

## Proteomes and PTMs in Phagocytosis During Fungi Invasion

Fungal pathogens and their hosts require rapid modulation of virulence and defense mechanisms. Fungal pathogens have developed rapid and precise gene expression, protein translation, and PTM regulation mechanisms in order to colonize, invade, and replicate during systemic infection, summarized in **Table 2** (Butler et al., 2009; Kronstad et al., 2011; Kronstad et al., 2012; Li H. L. et al., 2019; Bruno et al., 2020). In pathogens, virulence factors also evolved to resist host obstruction and interception, including capsules, melanin, biofilms, and growth at 37°C, among others (Cherniak and Sundstrom, 1994; Crabtree et al., 2012; Dambuza et al., 2018; Suo et al., 2018; Casadevall et al., 2019). To counter pathogenic invasion, host cells trigger a series of response cascades, restrict essential nutrients, produce cytokines and chemokines, induce infiltration of immune cells, and consequently activate eliminating mechanisms (Campuzano and Wormley, 2018; Casadevall et al., 2018).

**Table 2:** Summary of proteomic studies in host-fungal interaction.

Functions	Proteomics or PTMs	Pathogen and host	Description	Reference
Phagocytosis	proteomics	*C. albicans*, Human blood derived macrophages(M1 and M2 macrophage)	Characterized the proteomic differences between human M1 and M2 polarized macrophages in response to *C. albicans.*	(Reales-Calderon et al., 2014).
	proteomics, phosphorylation	*C. albicans*, RAW 264.7	Quantify macrophage proteins and phosphoproteins in RAW 264.7 exposed to *C. albicans.*	(Reales-Calderon et al., 2013).
	proteomics, phosphorylation	*C. albicans*, THP-1 macrophage	Quantitative proteomic and phosphoproteomic of human macrophage ATP-binding proteins exposed to *C. albicans*.	(Vaz et al., 2019)
	phosphorylation	*C. neoformans*, RAW264.7	Phosphoproteomic analysis of host response to *C. neoformans* infection in murine macrophage.	(Pandey et al., 2017)
	proteomics, lipidomics, and metabolomics	*C. neoformans*, murine bone marrow-derived macrophages and macrophages derived from human monocytes	Combination of proteomics, lipidomics, and metabolomics to investigate the roles of EVs from infected murine bone marrow-derived macrophages and macrophages derived from human monocytes interaction with *Cryptococcus*.	(Zhang L. et al., 2021)
	proteomics	*C. neoformans*, mouse	Comparison of transcriptome and proteome in lung tissues of *C. neoformans*-infected mice.	(Li et al., 2020)
	proteomics	*A. fumigatus*, RAW 264.7	Comparative proteomic analysis of mouse macrophage phagolysosomes containing melanized wild-type or nonmelanized pksP mutant conidia.	(Schmidt et al., 2018)
Energy Metabolism	proteomics	*C. albicans* and serum	Time-course proteomics in *C. albicans* in the presence or absence of FBS.	(Aoki et al., 2013a; Aoki et al., 2013b)
	proteomics, phosphorylation	*C. albicans*, THP-1 macrophage	Quantitative proteomic and phosphoproteomic of human macrophage ATP-binding proteins exposed to *C. albicans*.	(Vaz et al., 2019)
	proteomics	*A. fumigatus*, A549	Characterized the proteomic response of A549 exposed to *A. fumigatus*	(Margalit et al., 2020)
	proteomics	*C. gattii*, rat	Identify differentially expressed proteins induced by a *C. gattii* in a rat model by a shotgun proteomics	(Rosa et al., 2019)
	acetylation	*C. neoformans*, mouse	Comparative acetylome analysis in mouse model during *C. neoformans* infections	(Li et al., 2020)

Immunohistochemical staining, quantitative polymerase chain reaction, western blot, transcriptome analysis, and proteome and PTM analyses have provided valuable information on interactions between hosts and invading fungi. Phagocytosis by macrophages and glucose metabolism play important roles in interactions between pathogens and hosts (Rohatgi and Pirofski, 2015; Hansakon et al., 2019). The infection process is a complex of interactions between pathogen and host at RNA, protein, PTM, and metabolic levels. When a host was invaded, phagocytosis by macrophages clears invading pathogens (Li H. L. et al., 2019; Sun et al., 2019; Nelson et al., 2020; Seoane et al., 2020). Many studies show that phagosomes have a fundamental and distinct role in fungal infections (Sorrell et al., 2016; Santiago-Tirado and Doering, 2017; Santiago-Tirado et al., 2017; Li H. L. et al., 2019; Giusiano, 2020; Scherer et al., 2020), with phagocytosis regulated by both protein and PTM levels. Reales‐Calderón characterized the proteomic differences between human M1 and M2 polarized macrophages in both basal conditions and in response to *C. albicans*. They identified metabolic routes and cytoskeletal rearrangement components as the most relevant differences between M1 and M2. In addition, the switch from M1 to M2 may contribute to *C. albicans* pathogenicity by decreasing generation of specific immune responses or as part of a host attempt to reduce inflammation and limit damage from infection, which would increase fungal survival and colonization (Reales-Calderon et al., 2014). Reales-Calderon et al. (2013) used MS to quantify macrophage proteins and phosphoproteins in murine macrophages cell line RAW 264.7 exposed to *C. albicans*. They identified 68 differentially expressed macrophage proteins and 196 differentially abundant phosphorylation peptides, which altered pathways associated with receptors, mitochondrial ribosomal proteins, cytoskeletal proteins, and transcription factor activators involved in inflammatory and oxidative responses and apoptosis. The results suggested that apoptosis is a central pathway in the immune defense against *C. albicans* invasion (Reales-Calderon et al., 2013). Recently, Vaz et al. (2019) used a quantitative proteomic and phosphoproteomic approach to study human macrophage ATP-binding proteins exposed to *C. albicans*. They identified 59 differentially abundant ATP binding proteins, including 6 kinases (MAP2K2, SYK, STK3, MAP3K2, NDKA, and SRPK1), consistent with previous studies (**Figure 1G**) (Hole and Wormley, 2016; Ballou and Johnston, 2017; Vaz et al., 2019). Similar to *C. albicans*, in the initiation of *C. neoformans* infections, macrophages are the main phagocytic cells, and M1 macrophages can effectively inhibit pathogen spread. Nevertheless, *C. neoformans* can survive and reproduce inside macrophages. Consequently, macrophages can be a niche for pathogens to survive and spread. Pandey et al. (2017) found that host autophagy initiation complex (AIC), which regulates fungal colonization of mice, was regulated through kinase activities of upstream regulatory components of AIC, LKB1 and AMPKα1. Their discovery was based on a global phosphoproteomic analysis of host response to *C. neoformans* infection in murine macrophage cells (RAW264.7) using semi-quantitative, label-free nano liquid chromatography-MS/MS. They identified 1,268 differentially phosphorylated host proteins deemed responsive to *C. neoformans* (1.5 fold-change), which indicated a reprograming of host kinase pathways, especially in the AIC. Knockout of AMPKα1 in monocytes of mice results in resistance to fungal colonization (**Figure 1H**) (Pandey et al., 2017). To further understand the interaction between *C. neoformans* and macrophages, Zhang L. et al. (2021) used a combination of proteomics, lipidomics, and metabolomics to investigate the roles of EVs from infected murine bone marrow-derived macrophages and macrophages derived from human monocytes in the interaction with *Cryptococcus*. Pathway-associated p53, cell cycle and division, extracellular matrix receptors, and phosphatidylcholine were significantly enriched (Zhang L. et al., 2021). Consistent with *in vitro* investigations above, Li et al. (2020) compared the transcriptome and proteome in lung tissues of *C. neoformans*-infected C57BL/6J mice. They found a distinct set of differentially expressed genes and similar gene ontology (GO) and Kyoto Encyclopedia of Genes and Genomes (KEGG) enrichment analyses, which may be the result of different levels of PTMs (Li et al., 2020). Host phagosomes and energy metabolism are regulated at the pathogen–host axis at proteome and PTM levels and may play important roles during antagonistic interactions (Pandey et al., 2017; Schmidt et al., 2018; Li H. L. et al., 2019; Rosa et al., 2019; Sim et al., 2019; Sun et al., 2019; Vaz et al., 2019; Li et al., 2020; Nelson et al., 2020; Seoane et al., 2020). With the human fungal pathogen *A. fumigatus*, Schmidt et al. (2018) conducted a comparative proteomic analysis of mouse macrophage phagolysosomes containing melanized wild-type or nonmelanized *pksP* mutant conidia (Schmidt et al., 2018). Bioinformatical analysis of differentially expressed proteins revealed enriched pathways included vATPase-driven phagolysosomal acidification, Rab5 and Vamp8-dependent endocytic trafficking, and recruitment of Lamp1 phagolysosomal maturation marker and lysosomal cysteine protease cathepsin Z. Particularly notable, the proteome of invading *A. fumigatus* contained 22 differentially expressed proteins. Most importantly, the distinct roles of macrophages during fungal infections in humans remain to be confirmed.

## Host Energy Metabolism in Host-Fungi Interactions

Energy metabolism, especially glucose and fatty acid metabolism, plays critical roles at the pathogen–host axis at both RNA and protein levels (**Table 2**) (Li H. L. et al., 2019; Li et al., 2020). Glucose is a primary factor in the competition between host and invading pathogen, and its metabolism is critical for fungal survival (Idnurm et al., 2007; Li H. L. et al., 2019). In fungal pathogens, adaption to a nutritionally deficient environment is also a key factor in pathogenicity. In *C. albicans*, carbon sources influence biofilm formation and drug resistance by regulating cell wall components and those of the secretome, including adherence and pheromone-regulated proteins (Ene et al., 2012). According to time-course proteomics in yeast nitrogen base ± Fetal Bovine Serum (FBS) media, pathways associated with transport, detoxification, energy metabolism, and iron acquisition were enriched in *C. albicans* (Aoki et al., 2013a; Aoki et al., 2013b). Furthermore, Li H. L. et al. (2019) found that compared with *in vitro C. neoformans* results, *in vivo* glycolysis and TCA cycle pathways varied in *C. neoformans* isolated from both mouse and monkey infection models (Li H. L. et al., 2019). From the host aspect, as mentioned before, Vaz et al. (2019) used a quantitative proteomic and phosphoproteomic approach to study human macrophage ATP-binding proteins during *C. albicans* infections. They found significantly altered ATP and macrophage mitochondrial proteins, indicating energy metabolism of phagocytosis was also altered during *C. albicans* infections. Margalit et al. (2020) characterized the proteomic response of A549 exposed to *A. fumigatus* and identified changes in mitochondrial activity and energy output (Margalit et al., 2020). Rosa et al. (2019) used a shotgun proteomics approach to identify differentially expressed proteins induced by a *C. gattii* clinical strain in a rat model and found a potential Warburg-like effect (Rosa et al., 2019). Briefly, rat lungs were isolated for three days post incubation with avirulent and virulent *C. gatti* strains and then analyzed by MS/MS. Infection by *C. gattii* induced a dramatic change in protein expression, especially that of proteins related to energy metabolism, such as those involved in the aerobic glycolysis cycle, TCA cycle, and pyrimidine and purine metabolism. These results indicated *C. gattii* infection triggers important changes in energy metabolism that lead to activation of glycolysis and lactate accumulation, culminating in a cancer-like metabolic status known as the Warburg effect. Li et al. (2020) found similar results in mouse lung tissues at day seven postinfection with *C. neoformans*. They performed acetylome analysis and found that the reactome of differentially expressed Kac proteins primarily included those involved in glucose and fatty acid metabolism (Li et al., 2020). Because of the important roles of energy during infection progression, glucose metabolism and mitochondrial function have gradually become the focus of research in infectious diseases, from both host and pathogen aspects. Deciphering the mechanisms of co-evolution at fungi-host axis, which deserves more attention, will contribute the therapy for fungal diseases and development of novel anti-fungal drugs.

## Questions and Outlook

This literature review summarizes the many applications of MS-based proteome and PTM analyses that have increased understanding of fungal pathogenesis and interactions between pathogens and hosts. With increases in MS throughput and precision, proteomics is now widely used in the life sciences. Much has been learned using standard fungal strains, including *C. albicans*, *A. fumigatus*, *C. neoformans*, and *C. auris*, and samples from infected animal models, including mice and rats and cell lines such as RAW264.7, A549, and THP-1. However, shortcomings remain in this area. First, human-relevant samples are limited to only those with monocytes or body fluids. Second, differences among clinical fungal strains or primary cell types and in specific organs/tissues are far too great to ignore. Third, interactions of proteomes and regulation mechanisms among PTMs are poorly understood. In addition, although proteomics together with other omics can serve as comprehensive displays of cellular transcriptional levels, unfortunately, most multiomic studies are presented without simultaneous analyses and functional experiments (Zamith-Miranda et al., 2019; Zhou et al., 2021). This lack of supporting studies may be due to constraints with database integration and interconnectivity of omics data (Song M. et al., 2020). Over the past decade, a series of multiomics tools and data sets have proven to be valuable. However, simultaneously, higher requirements have become necessary for data operation, and computational resources, ethical regulatory issues associated with data sharing, application of machine and deep learning, and development of data visualization tools need to be addressed (Krassowski et al., 2020). With the advent of the big data era, combined multiomics is expected to be a very powerful tool in future research on pathogenic fungi. Furthermore, there is a great potential to improve MS techniques, particularly to increase detection resolution. In addition, dual-proteome or dual-PTM analyses of pathogens and host are difficult to conduct and need to be improved. In the future, mass spectrometry will be used to identify important proteins, PTMs, and their functions in the fungi and fungi-host interaction repertoire, and benefits for fungal therapeutics and vaccine development. Overall, MS is a novel approach that will continue to help decipher mechanisms of fungal diseases. Understanding fungal pathogenesis and clinically relevant interactions between host and fungal strains contributes to the development of novel clinical therapies and antifungal drugs and helps to identify clinical biomarkers to combat deadly fungal infections and decrease morbidity and mortality.

## Author Contributions

Writing—original draft preparation: TS, HL, YL, and CD. Writing—review and editing: CD. All authors contributed to the review and approved the submitted version.

## Funding

This review was supported by the National Natural Science Foundation of China (31870140 to CD and 81801989 to TS), the Liaoning Revitalization Talents Program (XLYC1807001 to CD), the Beijing Natural Science Foundation (5184037 to TS), the Fundamental Research Funds for the Central Universities (3332018024 to TS), and the China Postdoctoral Science Foundation (2021M693520 to HL).

## Conflict of Interest

The authors declare that the research was conducted in the absence of any commercial or financial relationships that could be construed as a potential conflict of interest.

## Publisher’s Note

All claims expressed in this article are solely those of the authors and do not necessarily represent those of their affiliated organizations, or those of the publisher, the editors and the reviewers. Any product that may be evaluated in this article, or claim that may be made by its manufacturer, is not guaranteed or endorsed by the publisher.
